# A guideline for the prevention and management of Fetal Alcohol Spectrum Disorder in South Africa

**DOI:** 10.1186/s12913-019-4677-x

**Published:** 2019-11-06

**Authors:** Babatope O. Adebiyi, Ferdinand C. Mukumbang, Anna-Marie Beytell

**Affiliations:** 1 School of Public Health, University of the Western Cape, Cape Town, 8001 South Africa; 2 Department of Social Work, University of the Western Cape, Cape Town, South Africa

**Keywords:** Fetal alcohol spectrum disorder, Policy, Guideline, Delphi approach, Management, Prevention, Snowball sampling, Developmental disabilities, Women

## Abstract

**Background:**

Fetal Alcohol Spectrum Disorder (FASD) is a public health problem globally, with South Africa having the highest recorded prevalence of all countries. Government programmes to prevent and manage FASD remain limited because of the lack of a specific policy. Herein, we developed a guideline to inform policy on the prevention and management of FASD in South Africa.

**Methods:**

We applied a modified version of the World Health Organization’s approach to guideline development in three phases. In the first phase, we designed the initial guideline prototype. To do this, we conducted an in-depth interview with policymakers and a focus group with relevant service providers on policy requirements for FASD, a document review of policies on FASD and a scoping review of various interventions for FASD. In phase 2, we refined the initially formulated guideline prototype through a discursive approach with seven local and international experts on FASD. Phase 3 involved refining the prototype using a modified Delphi approach. Forty-three and forty-one experts participated in rounds 1 and 2 of the Delphi approach, respectively. The acceptable consensus for each included policy statement was 85%.

**Results:**

We identified three aspects of the proposed guideline, which are the approaches and guiding principles, the prevention measures and the management measures. The guideline proposes that a FASD policy should consider lifespan needs, be culturally diverse, collaborative, evidence-based, multi-sectoral and address social determinants of health contributing to FASD. The essential components of FASD prevention policy consist of awareness and education of the dangers of drinking alcohol, access to treatment for alcohol problems and training of service providers. The management components include capacity building related to diagnosis, educating parents regarding the needs and management, appropriate referral pathways, training of teachers regarding classroom management and support for parents and individuals with FASD.

**Conclusion:**

FASD in South Africa deserves urgent attention. Developing a specific policy to guide programmes could enhance and coordinate the efforts towards preventing and managing FASD. The guideline has the potential to assist policymakers in the development of a comprehensive and multi-sectoral policy for prevention and management of FASD, considering the consensus obtained from the experts.

## Contributions to the literature

- How to adapt WHO’s approach for guideline development to develop a guideline for policy.
- How to develop/improve a guideline/policy using a multi-method study with a modified Delphi approach.
- Policymakers’, service providers’ and experts’ perspectives regarding policy requirements for the prevention and management of FASD.
- The essential components of FASD policy, which include awareness and education of the dangers of drinking alcohol, access to treatment for alcohol problems, training of service providers, capacity building related to diagnosis and support for parents and individuals with FASD.

## Background

Globally, one in every 13 alcohol-exposed pregnancies results in a Fetal alcohol spectrum disorder (FASD) [1]. In 2017, the global prevalence of FASD was 8 per 1000 children and youth [1]. In 2016, the Foundation for Alcohol Related Research (FARR), estimated that 6 million individuals were affected by FASD in South Africa [2] ranging from 29 to 290 per 1000 live births, the highest recorded prevalence in any part of the world [3]. The prevalence of FASD varies from one province to another, with the Western Cape and Northern Cape provinces the most affected. In 2017, the prevalence of FASD among Grade 1 pupils in the Western Cape was estimated to be 196 to 276 per 1000 [4], while in the Northern Cape the prevalence was estimated at 63.9 per 1000 Grade 1 pupils in 2015 [5].

Although FASD can be clinically identified through characteristic facial features that can be observed on examination [6, 7], some FASD are considered as hidden disabilities because in most cases there are no noticeable physical manifestations [8–11] thus increasing the chances of missed diagnosis or misdiagnosis [12–15]. Misdiagnosis and missed diagnoses prevent individuals from accessing early and appropriate services [16]. To diagnose any of the FASD (fetal alcohol syndrome {FAS}, partial FAS, {PFAS}, alcohol-related neurodevelopmental disorder {ARND} and alcohol-related birth defects {ARBD}), individuals must meet all or some of the range of identified criteria [17–19]. These criteria include documented or undocumented prenatal alcohol exposure, prenatal and or postnatal growth deficiency, deficient brain growth and neurobehavioral impairment – with or without cognitive impairment [17–19]. The requirements for prevention, diagnosis and management suggests a multi-sectoral approach [3, 9, 20–23].

While the South African government endeavours to address the high prevalence of FASD, its responses through policy have not been adequate [24, 25]. Despite the availability of context-relevant evidence-based interventions such as case management and universal prevention approaches to prevent FASD [26, 27], there is a lack of policy and resources to guide the expansion and implementation of these interventions to many parts of the country. There is evidence that current prevention and management interventions are informed by genetic-related and other generic policy documents [21, 28]. For instance, the Human Genetics Policy Guidelines for the Management and Prevention of Genetic Disorders, Birth Defects and Disabilities and the Mini Drug Master Plan covers FASD as a genetic disease. The National Department of Health also recognises FASD as one of the ten focal genetic conditions. The Western Cape government has listed FASD as a provincial health priority in its services regarding birth defects [29]. Because these documents reflect FASD in a generic manner, they do not support the holistic and comprehensive (multi-sectoral) approach required to address FASD [24, 25, 30].

In addition to the efforts of the South African national and provincial governments, non-profit organisations (NPO) provide various levels of contributions to address FASD in South Africa. The South African government provides partial funding to some of these organisations. These organisations function mainly in the Western Cape, though they have a presence in other provinces, especially Northern Cape and Eastern Cape. FARR, for example, provides services such as mentorship, creating social awareness, providing education and training programmes, conducting medical and psycho-social FASD-related research and providing support and diagnostic services [31]. FASfacts, as an NPO, offers FASD prevention programmes through experiential learning, advertising campaigns using churches, films and theatres to pass FASD messages and mentoring of pregnant women [32]. These organisations obtain funding from the government by aligning their programmes to the existing generic policies.

Different approaches have been proposed to develop relevant and effective FASD policies. One such approach proposed to prevent and manage FASD, is the decolonisation policy discourse [33]. The proponents of the decolonised policy discourse advocate that women should be seen as victims of the FASD problem, not as perpetrators [33]. They also propose that the socio-economic and socio-political circumstances that predispose women to alcohol consumption during pregnancy, which may lead to FASD, should be addressed [33]. Other proposed approaches are the comprehensive (addressing all factors); inclusive (involving all sectors and all levels of the government); and the human rights-based approach (acknowledging the principles of non-discrimination, participation, inclusion, equity and access) [34]. A women-centred approach, which considers the needs of women in all aspects of design and delivery, including the location and accessibility of services, staffing, programme development, content and materials has also been proposed [34]. We propose that for an FASD policy to be truly comprehensive and multi-sectoral, all the above approaches should be scrutinised.

Having a multi-sectoral and comprehensive policy [21, 35] as demonstrated by the Canadian and Australian governments [34, 36, 37], has the potential to coordinate existing and new approaches and programmes for preventing and managing FASD. In South Africa, government programmes to address FASD remain limited partly because of the absence of a policy to inform prevention and management [29]. To this end, we sought to develop a guideline to inform the design of a comprehensive and multi-sectoral policy to address FASD in South Africa [30].

## Methods

**Fig. 1:**
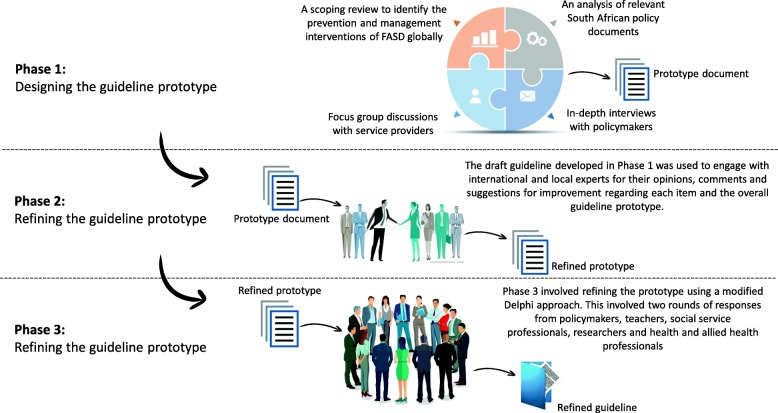
Study design approach

In developing the guideline for the prevention and management of FASD, we adapted the World Health Organization’s approach (steps) [38, 39], which we organised in three phases (Fig. 1). The first phase entailed designing the initial guideline prototype. In Phase 2, we refined the initially formulated guideline prototype. Phase 3 involved testing and confirming the refined prototype using a modified Delphi approach.

### Phase 1: designing the guideline prototype

We conducted focus group discussions with service providers and in-depth interviews with policymakers to identify policy requirements for FASD in South Africa [25, 30]. The service providers included educators, health and allied health professionals as well as social service professionals providing services to women, especially pregnant women or individuals with FASD or related conditions. The policymakers included individuals working in the Departments of Social Development, Health and Education on FASD-relevant issues or related conditions. The service providers and the policymakers were asked various questions regarding current practices and interventions and policy requirements for FASD.

In addition to the focus group discussions and in-depth interviews, we conducted a document review. We identified clauses of FASD policy in various South African-related policy documents [28]. We searched databases (PubMed and Google search engines) and the websites of South African national and provincial departments. We used the following search terms: foetal alcohol spectrum disorder, alcohol-related neurodevelopmental disorder, foetal alcohol syndrome, white paper, green paper, policy, action plan, gazette and South Africa. Although, we used “foetal” for the search, it includes documents with “fetal”. We also contacted the designated persons in the departments of Education, Health, Social Development and Trade and Industry via emails to request other relevant documents.

Furthermore, we conducted a scoping review to identify the prevention and management interventions of FASD globally that could be included in the policy [38]. We searched the following Ebsco Host embedded databases: Academic Search Complete, ERIC, SoINDEX, Health Source. We also searched the Nursing/Academic Edition, CINAHL, Medline and Psych-ARTICLES, Springer Links, SAGE Journals and PubMed databases. The search terms used include fetal alcohol spectrum disorder, fetal alcohol syndrome, alcohol-related neurodevelopmental disorder, alcohol-related birth defects, partial fetal alcohol syndrome, prenatal alcohol exposure, intervention, strategy, treatment, programme, management, prevention and therapy.

**Fig. 2:**
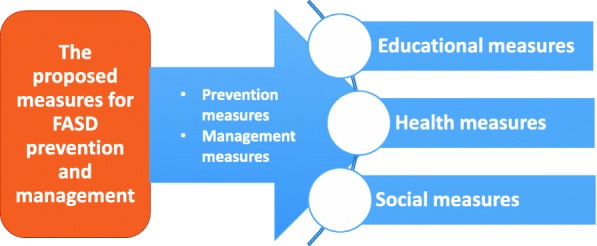
Heuristic classification framework applied to summarise the data

Data obtained from the various sources were analysed using content and thematic framework analyses in the different studies. The findings from these studies were integrated towards developing the initial guideline prototype using the framework indicated in Fig. 2.

The information gleaned from the various sections as represented in the framework were then rephrased into different statements to formulate a draft guideline.

### Phase 2: refining the initial guideline prototype

The draft guideline developed in Phase 1 was used to engage with seven international and local experts **(**Table 1**)** for their opinions, comments and suggestions for improvement regarding each item and the overall guideline prototype. These experts were purposely sampled based on their availability and expertise. Emails were sent to them requesting their participation. We selected those who had experience in conducting FASD research and those who had been involved in developing FASD policies.

**Table 1:** Characteristics of international and local experts

Type of expert	Sex	Position
International	Female	Professor
International	Female	Researcher
International	Female	Policymaker
International	Male	Researcher
Local	Male	Professor
Local	Female	Researcher
Local	female	Lecturer

Following the engagement with the experts, we obtained a refined guideline prototype **(**Table 2**)**.

**Table 2:** The prototype guideline for the prevention and management of the FASD policy

Approaches and guiding principles and approaches – the proposed FASD policy should be…	
Holistic	
User- and caregiver-focused	
Inter-departmental/multi-sectoral	
Considerate of needs across the lifespan	
Collaborative	
Human rights-based	
Based on a public health framework	
Culturally diverse and culturally sensitive	
Evidence-based	
Woman/family-centred	
Clear about referral pathways	
Designed to avoid victim blaming	
Cost-effective	
Driven by behavioural economics	
Education-related proposed prevention measure for FASD – the proposed FASD policy should…	
Enhance awareness of the dangers of drinking alcoholic beverages during pregnancy in schools including colleges and universities	
Assist individuals with alcohol-use problems in educational settings to access treatment	
Address barriers to access treatment for alcohol-related problems in educational settings	
Address stigma associated with alcohol abuse in educational settings	
Facilitate training of teachers re the FASD prevention/awareness programme	
Facilitate the development and implementation of FASD awareness programmes in schools (including colleges and universities)	
Facilitate the use of peer education for the FASD awareness programme in schools	
Promote a healthy lifestyle in schools through sport and other extra-curricular activities	
Make school events alcohol-free	
Discourage the establishment of liquor stores in the proximity of schools	
Promote the education of young individuals about healthy pregnancy in schools	
Health-related proposed prevention measures for FASD – should…	
Facilitate screening for alcohol use in clinics and hospitals	
Encourage proper documentation of the alcohol history of women, especially pregnant women	
Facilitate the inclusion of FASD prevention as a part of health promotion activities in clinics and hospitals	
Facilitate the education of individuals and couples re the dangers of drinking alcoholic beverages during pregnancy in the pre-conception clinic	
Facilitate the education of individuals and couples re the dangers of drinking alcoholic beverages during pregnancy in the reproductive clinic	
Encourage the use of visible posters and pamphlets for FASD prevention campaigns in all clinics and hospitals	
Facilitate training of healthcare professionals re FASD prevention	
Facilitate early and appropriate referral to treatment for individuals (including women) with alcohol misuse issues	
Empower health professionals with the skills to counsel and ask questions about safe and appropriate alcohol use	
Promote the use of contraceptives to avoid unplanned pregnancy	
Community/social-related proposed prevention measures for FASD – should…	
Facilitate public awareness re the dangers of alcohol abuse	
Facilitate the education of all people in the community re the dangers of drinking alcohol during pregnancy	
Facilitate the education of individuals and couples re the dangers of drinking alcohol during pregnancy	
Encourage the use of community groups for FASD prevention (education and awareness)	
Facilitate the training of the community health/community-based workers and youth care/social workers re FASD prevention	
Facilitate early intervention and assistance for individuals with alcohol-use problems in the community	
Facilitate the creation of social programmes such as skills training and empowerment programmes for women in the community	
Encourage awareness and education re FASD in the workplace, rural and urban areas and farming communities	
Promote the use of multimedia such as posters, adverts, pamphlets, TV, social media and road shows re FASD awareness in the communities	
Promote enforcement of liquor laws and regulation of shebeens to control accessibility and availability of alcohol in the community	
Provide access to treatment for people with alcohol-use problems in the community	
Provide smooth aftercare and community reintegration to people who have attended alcohol rehab	
Promote afterschool activities in the community to prevent early exposure of adolescents to alcohol	
Discourage all advertisements that link alcohol to sport/other popular community events/activities	
Mandate labels on alcohol containers to contain information re the dangers of drinking alcoholic beverages during pregnancy	
Mandate that liquor stores display warning signs regarding alcohol and pregnancy	
Enable the creation of support groups for individuals with alcohol misuse issues in the community	
Facilitate the training of the community and religious leaders re FASD prevention	
Promote collaboration and the use of non-profit organisations (NPO) re FASD prevention	
Utilise community and religious leaders to increase FASD awareness among their communities	
Promote the expansion and adoption of NPO evidence-based interventions re prevention in the community	
Assist families to support individuals with alcohol-use problems	
Education-related proposed management measures for FASD – should...	
Facilitate the development of a curriculum that accommodates individuals with FASD	
Facilitate training of teachers re the classroom management for individuals with FASD	
Promote skilled schools for learners with learning disabilities (including individuals with FASD) who are not benefiting from formal education	
Make provision for special assistance for individuals with FASD within mainstream schools	
Facilitate the creation of the special schools for learners with a learning disability (including individuals with FASD) who are not benefiting from mainstream schooling	
Facilitate the education of parents re the needs and management of individuals with FASD	
Health-related proposed management measures for FASD – should...	
Facilitate capacity building re diagnosis among health professionals	
Facilitate FASD screening for all children who are known to have been prenatally exposed to alcohol	
Make provision for diagnostic services for individuals	
Promote diagnosis for school children, adolescents and adults to reduce rates of people who are left undiagnosed or misdiagnosed	
Promote appropriate referral pathways to services after diagnosis	
Facilitate the creation of diagnostic centres in clinics, hospitals and communities	
Facilitate the creation of national surveillance for FASD via reports from health professionals	
Make provision for integrated and individualised medical services for individuals with FASD	
Encourage routine consideration of FASD in the diagnosis and management of mental illness and developmental disorders	
Community/social-related proposed management measures for FASD – should...	
Provide skills training and empowerment programmes for those in need among individuals with FASD	
Facilitate appropriate employment opportunities for individuals with FASD	
Facilitate the training of community health workers/community-based workers/youth care workers/social workers and professionals within judiciary system re FASD management	
Facilitate the training of the biological and foster parents/caregivers regarding the management of FASD	
Promote the empowerment of the parents/caregivers of individuals with FASD in the community	
Promote the establishment of support systems for biological and foster parents/caregivers and individuals with FASD in the community	
Promote the referral of parents and individuals with FASD to appropriate services	
Make provision for effective counselling services for parents and individuals with FASD	
Encourage family/community support for individuals with FASD	
Provide support for individuals with FASD in child protection/foster care and the criminal justice system	
Facilitate the creation of a structure and supportive environment at home, school and beyond	

### Phase 3: testing and finalising the guideline

We conducted a modified Delphi approach to improve the prototype guideline developed for policy on FASD.

#### Development of questionnaire

The statements in the refined prototype developed in Phase 2 **(**Table 2**)** were used to design Likert scale statements for the Delphi responses. We asked the respondents to rate their agreement with each statement on a 5-point Likert scale (from ‘strongly disagree’ to ‘strongly agree’) whereby they could answer ‘neutral’ when they were not sure how to rate a statement. The questionnaire was divided into three sections (principles and approaches to policy development, prevention considerations and management consideration) and three sub-sections (education, health and social aspects). The participants were encouraged to provide further comments at the end of each section and sub-section. The questionnaire was piloted among international and local experts working on the prevention and management of FASD to ensure coherence, feasibility and validity.

#### Recruitment of the participants

Using purposive and snowball sampling techniques, we recruited professionals who have experience and or expertise on FASD. First, we purposively recruited 15 participants (policymakers, teachers, social service professionals, researchers and allied health professionals) as the seed participants. We used the following criteria for selecting the first batch of participants (i) having experience working with women (especially pregnant women) and or individuals with FASD or related conditions, (ii) experience in making or implementing FASD policy or related conditions, and (iii) having published articles or conducted FASD research or related conditions. Second, we asked the 15 participants to invite others in their networks (snowballing). Through purposive and snowball approaches, 43 participants completed round 1 of the Delphi study. We invited the 43 respondents who participated in round 1, and 41 completed the round 2 questionnaire. In Table 3, the characteristics of the participants in both rounds of the Delphi study are shown.

**Table 3:** Characteristics of the participants

Characteristics	Round 1(*n* = 43)	Round 2 (*n* = 41)
Gender		
Male	11	11
Female	32	30
Occupation		
Social services provider	14	13
Researcher	9	9
Policymaker	12	11
Allied health and health	5	5
Teacher	3	3
Highest level of education		
High school	1	1
College	3	3
University	39	37
Years of experience		
1–5	14	13
6–10	12	11
11–15	7	7
16 and above	10	10

#### Data collection

We created a Google form, which allowed the participants to respond online and invited them via email to follow the link to this form. Participants were given an initial period of 2 weeks to respond to the questionnaire. The 2 weeks were extended to 2 months to increase participation including the participants recruited by snowballing. We reminded those who had not responded before the end of the 2 weeks as well as weekly up to the 2 months. The round 1 questionnaire allowed participants not only to agree or disagree with a set of statements but also to provide comments to improve the guideline. Before we administered the questionnaire, we agreed that if a statement reached at least 85% consensus from the participants, it would be endorsed. Statements not reaching the 85% consensus, and the new ones generated from the analysis of the comments of the participants in round 1, were prepared as a separate questionnaire and administered in round 2.

#### Validity

To ensure validity in this study, we consulted with local and international experts to ensure the content covered all the areas of the variable being measured. The experts’ opinions were used to improve the guideline before it was used for the Delphi study. We also consulted questionnaires and surveys designed to measure similar concepts.

#### Reliability

To ensure reliability, we asked some of the participants to answer the questionnaire for a second time. Both their responses were compared to show consistency. Additionally, we calculated Cronbach’s alpha for the study using the Statistical Package for Social Science (SPSS) software. The Cronbach’s alpha for round 1 is 0.977, and round 2 is 0.796.

#### Data analysis

We analysed the quantitative data using SPSS, generating descriptive statistics (frequencies) for each statement.

### Ethical considerations

The research ethics committee of the University of the Western Cape approved this study (BM/16/4/4). Approvals were also obtained from the Western Cape Department of Education (20161212–6937), Departments of Health (WC_2016RP29_862) and Social Development (12/1/2/4). Eligible experts were invited to participate via email with the inclusion of an information sheet and a consent form. The experts were required to read the information sheet to understand the purpose of the study and what they needed to do if they agreed to participate. Those who agreed to participate were asked to sign the consent form. We maintained confidentiality during the research by anonymising the participants.

## Results

### Guiding principle and approach

Three of the statements achieved 100% consensus in round 1 and none in round 2 **(**Additional file 1a). Women/family-centeredness attained 84% consensus in round 1 and based on the comments made by some of the participants, we decided to separate family from women in round 2. The participants said having women-centred as one of the approaches and guiding principle could promote stigma and blame games. In round 2, family-centredness reached 95% consensus while women-centredness only achieved 68% agreement. However, the policy should be designed to avoid gender-focused interventions recorded the lowest consensus of 66%. There was no particular reason given by the participants for the latter.

### Proposed prevention measure

#### Education-related proposed prevention measure

In round 1, only one of the statements attained 100% agreement from the respondents with none in round 2. However, most statements achieved 90% and greater in terms of agreement. The statement, ‘Make school events alcohol-free’ attained the lowest agreement at 81%. When administered in round 2, it only obtained an agreement rate of 78% **(**Additional file 1b).

#### Health-related proposed prevention measures

Five of the statements on health-related proposed prevention measures reached 100% consensus among participants in round 1 **(**Additional file 1c). The only statement generated from the comments made by participants in round 1 and administered in round 2 attained 93% agreement.

#### Community/social-related proposed prevention measures

Eight statements had 100% consensus in round 1 while one attained 100% agreement in round 2. The remaining statements achieved agreement in 90% and above except two (statements 14 and 23). ‘Discourage all advertisements that link alcohol to sport/other popular community events/activities’ was re-administered in round 2 and recorded a lower consensus (71%) than round 1. Some of the participants commented that a ban on the advertisement has little or no impact on reducing or preventing alcohol consumption during pregnancy in an area where drinking is rampant **(**Additional file 1d).

### Proposed management measures

#### Education-related proposed management measures

Of the six education-related proposed measures, only one attained 100% of agreement in round 1. The remaining statements also achieved a consensus rate of 90% and above (Additional file 1e). Therefore, none of the statements was administered in round 2.

#### Health-related proposed management measures

None of the health-related proposed management measures achieved 100% agreement in both rounds (Additional file 1f). Five of the statements reached consensus at 90% and above. The remaining statements attained 85% or more consensus, except one. Although all the statements in this sub-section reached the acceptable consensus for this study, some of the participants raised concerns. They commented on the feasibility and practicality of diagnosing school children, adolescents and adults, individualised medical services and creating national surveillance. They cautioned that it is not advisable to diagnose without making provision for services after diagnosis. They reported that individualised services would be too expensive and appropriate logistics have to be implemented for national surveillance. In round 2, we decided to separate integrated and individualised medical services. Only integrated service reached the consensus (88%) acceptable in this study.

#### Community/social-related proposed management measures

None of the statements attained 100% consensus in round 1, whereas one reached 100% agreement in round 2 (Additional file 1g). Besides one statement, all the others achieved a consensus of 90% or more. The promotion of grant/social welfare for individuals with FASD attained the lowest consensus (49%). One of the reasons given for the low consensus was that promoting grants and social welfare would encourage women to drink during pregnancy to receive these benefits.

Following the two rounds of responses in the Delphi process, we selected only those statements that qualified by 85% in either round to be included in the guideline **(**Table 4**).**

**Table 4:** A proposed guideline for policy on the prevention and management of FASD.

Overall guiding principles of the policy	
The panel agreed that policy to inform the prevention and management of FASD should	
• Be holistic, considering the prevention, diagnosing and management of FASD.	
• Consider the individuals with FASD and their caregivers.	
• Involve all relevant government departments such as the departments of health, education, justice, social development, trade and industry, labour.	
• Consider the needs of individuals with FASD throughout their lifespan.	
• Involve the collaborative action of various professionals (social service, justice and healthcare); healthcare professionals from the doctors, midwives, nurses, to the community healthcare workers.	
• Be holistic, considering the prevention, diagnosing and management of FASD.	
• Adopt a human rights-based approach, which protects and promotes the rights of women, children, families and communities affected by FASD and recognises the principles of non-discrimination, participation, inclusion, equity and access.	
• Adopt a public health framework, which acknowledges drinking during pregnancy and FASD are part of a complex interplay of biological, social, psychological, environmental and economic factors.	
• Be culturally diverse and culturally sensitive, acknowledging the importance and strength of cultural values and norms.	
• Use relevant and current evidence to inform practice and interventions to strengthen the knowledge base to effectively prevent and manage FASD.	
• Establish clear referral pathways for the effectiveness of the prevention and management FASD	
• Avoid victim blaming that is placing women at the centre of the FASD problem, which will not consider them as the perpetrators of the problem.	
• Consider cost-effective interventions, which encourages a population-wide approach and enhances a wider coverage.	
• Be family-centred, service providers must acknowledge and value the need for individuals within the family structure.	
• Address social determinants of health contributing to FASD.	
• Consider input from individuals with FASD and their families in developing a policy for the prevention and management of FASD.	
• Promote responsible parenting, which recognises human values and enhance the development of individuals with FASD.	
FASD prevention measures	
Education-related prevention measures for FASD	
The panel agreed that an FASD policy on education-related prevention should contain strategies to	
• Increase awareness of the dangers of drinking alcoholic beverages during pregnancy in educational settings.	
• Assist individuals with alcohol-use problems in educational settings to access treatment.	
• Address the barriers to access treatment for alcohol-related problems in educational settings.	
• Address stigma associated with alcohol abuse in educational settings.	
• Improve training of teachers on FASD prevention/awareness programme.	
• Facilitate the development and implementation of FASD awareness programmes in educational settings.	
• Facilitate the use of peer education for FASD awareness programme in educational settings.	
• Promote healthy lifestyle in schools through sport and other extra-curricular activities.	
• Discourage the establishment of the liquor stores in the proximity of educational settings.	
• Promote the education of young individuals about healthy pregnancy in educational settings.	
• Facilitate the teaching of responsible parenthood in educational settings.	
• Improve the teaching of safe sex practices in educational settings.	
Health-related prevention measures for FASD	
The panel agreed that an FASD policy on health-related prevention should contain strategies to	
• Improve the screening of alcohol use in clinics and hospitals.	
• Improve documentation on the alcohol history for women, especially pregnant women.	
• Facilitate the inclusion of FASD prevention as a part of health promotion activities in clinics and hospitals.	
• Improve the education of individuals and couples on the dangers of drinking alcoholic beverages during pregnancy in the pre-conception clinic.	
• Improve the education of individuals and couples on the dangers of drinking alcoholic beverages during pregnancy in the reproductive clinic.	
• Encourage the use of visible posters and pamphlets for FASD prevention campaigns in all clinics and hospitals.	
• Improve the training of healthcare professionals on FASD prevention.	
• Improve early and appropriate referral to treatment for individuals (including women) with alcohol misuse issues.	
• Empower health professionals with the skills to counsel and ask questions about alcohol use in a safe and appropriate way.	
• Promote the use of contraceptives to avoid unplanned pregnancy.	
• Improve assistance to parents of individuals with FASD to avoid having another child with FASD.	
Community/social-related prevention measures for FASD	
The panel agreed that an FASD policy on community-related prevention should contain strategies to	
• Improve education and public awareness of the dangers of alcohol abuse.	
• Improve the education of all people in the community on the dangers of drinking alcohol during pregnancy.	
• Improve the education of individuals and couples on the dangers of drinking alcohol during pregnancy in the community.	
• Encourage the use of community groups for FASD prevention (education and awareness).	
• Improve the training of the community health /community-based workers and youth care/social workers on FASD prevention.	
• Facilitate early intervention and assistance for individuals with alcohol-use problems in the community.	
• Facilitate the creation of social programmes such as skills training and empowerment programmes for women in the community.	
• Improve awareness and education on FASD in the workplace, rural and urban areas and farming communities.	
• Promote the use of multimedia such as posters, adverts, pamphlets, TV, social media and road shows for FASD awareness in the communities.	
• Improve enforcement of liquor laws and regulation of shebeens to control accessibility and availability of alcohol in the community.	
• Improve access to treatment for people with alcohol use problems in the community.	
• Improve smooth aftercare and community reintegration for people who have attended alcohol rehab.	
• Promote afterschool activities in the community to prevent early exposure of adolescents to alcohol.	
• Mandate labels on alcohol containers to contain information on the dangers of drinking alcoholic beverages during pregnancy.	
• Mandate that liquor stores have warning signs regarding alcohol and pregnancy.	
• Enable the creation of support groups for individuals with alcohol misuse issues in the community.	
• Facilitate the training of the community and religious leaders on FASD prevention.	
• Promote collaboration and the use of non-profit organisation (NPO) for FASD prevention.	
• Utilise the community and religious leaders to increase FASD awareness among their communities.	
• Promote the expansion and adoption of NPO evidence-based interventions for prevention in the community.	
• Improve assistance to families to support individuals with alcohol use problems.	
• Improve interventions services for mothers who have a child with FASD in the community.	
FASD management measures	
Education-related management measures for FASD	
The panel agreed that an FASD policy on education-related management should contain strategies to	
• Facilitate the development of a curriculum that accommodates individuals with FASD.	
• Improve the training of teachers on classroom management for individuals with FASD.	
• Promote skill schools for learners with learning disabilities (including individuals with FASD) that are not benefiting from formal education.	
• Provide special assistance for individuals with FASD within mainstream schools.	
• Facilitate the creation of the special schools for learners with a learning disability (including individuals with FASD) that are not benefiting from mainstream schooling.	
• Facilitate the education of parents on the needs and management of individuals with FASD.	
Health-related management measures for FASD	
The panel agreed that an FASD policy on the health-related management should contain strategies to	
• Increase capacity building re diagnosis among health professionals.	
• Facilitate FASD screening for all children that are known to have been prenatally exposed to alcohol.	
• Provide diagnostic services for individuals.	
• Promote diagnosis for school children, adolescents and adults to reduce rates of people who are left undiagnosed or misdiagnosed.	
• Promote appropriate referral pathways to services after diagnosis.	
• Facilitate the creation of diagnostic centres in clinics, hospitals and communities.	
• Facilitate the creation of national surveillance for FASD via reports from health professionals.	
• Encourage routine consideration of FASD re the diagnosis and management of mental illness and developmental disorders.	
• Provide integrated medical services for individuals with FASD.	
Community/social-related management measures for FASD	
The panel agreed that an FASD policy on the community-related management should contain strategies to	
• Provide skills training and empowerment programmes for those who need it among individuals with FASD.	
• Facilitate appropriate employment opportunities for individuals with FASD.	
• Facilitate the training of community health workers/community-based workers/ youth care workers/ social workers and professionals within the judiciary system re FASD management.	
• Improve the training of the biological and foster parents/caregivers regarding the management of FASD.	
• Promote the empowerment of the parents/caregivers of individuals with FASD in the community.	
• Promote the establishment of support systems for biological and foster parents/caregivers and individuals with FASD in the community.	
• Promote the referral of parents and individuals with FASD to appropriate services.	
• Provide effective counselling services for parents and individuals with FASD.	
• Encourage family/community support for individuals with FASD.	
• Provide support for individuals with FASD in child protection/foster care and the criminal justice system.	
• Facilitate the creation of structure and supportive environment at home, school and beyond.	
• Facilitate the provision of adequate information about individuals with for the adoptive parents.	

## Discussion

In this study, we aimed to develop a guideline that could assist policymakers in designing a holistic, comprehensive and multi-sectoral policy toward the prevention and management of FASD in South Africa. We considered a guideline as a document that contains evidence-based recommendations for the prevention and management of FASD and systematically developed statements capable of guiding policymakers to develop a holistic policy. This guideline has the potential to assist South African policymakers in developing a policy that will address FASD considering the acceptable consensus (85%). Furthermore, it can be adapted or adopted to guide the development of policy for the prevention and management of FASD in other countries, especially within sub-Sahara Africa.

Our findings indicated good support for the proposed approaches and principles of FASD policy. This support confirms our proposition that policies designed to guide the prevention and management of FASD should be holistic, user- and caregiver-focused, culturally diverse and sensitive, considerate of needs across the lifespan, collaborative, have clear referral pathways, evidence-based and inter-departmental. These policies should also use the public health framework, and be human rights-based, inter-departmental, collaborative, culturally diverse and sensitive, evidence-based, cost-effective and family-centred approach [34, 36, 37]. If these perspectives are taken into consideration, the chances of obtaining a policy that enhances effective and sustainable programmes to prevent and manage FASD such as those developed in Australia and Canada are high [34, 36, 37].

While designing the initial guideline, based on the finding from previous phases, we considered adding that FASD policies should be women-centred. Nevertheless, the experts in the Delphi approach suggested that women-centeredness as a guiding principle has the tendency to promote stigmatisation and victim-blaming of women. Stigma may also influence the prevention and management of FASD as has been reported [9]. The opinions expressed by these participants are consistent with the current policy discourses to re-contextualise and decolonise FASD policies and reframe the problem of FASD in a way that avoids victim-blaming [33]. This principle supports the argument that drinking during pregnancy should not be criminalised [39]. Rather, women with alcohol problems should be assisted. Therefore, an FASD policy must address multiple factors (local and systemic) that predispose women to alcohol abuse [33]. An FASD policy should also address the social, structural and economic factors affecting health behaviour and examine the growing gap in health inequities [33]. Thus, the notion of having a policy which addresses upstream drivers (social determinants of health contributing to FASD) is in alignment with these arguments [40].

A FASD prevention policy should support the training of teachers for FASD prevention and awareness as well as classroom management and the modification of curriculum for the benefit of individuals with FASD in an educational setting. These findings are supported by studies conducted to examine the relational experience of educators and the education needs of children with FASD [41, 42]. The authors of these studies recommend specialised training for educators, empowering them to assist individuals with FASD in the classroom to maximise their potential [41, 42]. Adequately trained educators can address the challenges of FASD in the classroom [43]. Goal 2 and priority 3 of the Canadian framework for action on FASD and the Australian national strategy for FASD, respectively emphasised on capacity development [36, 37]. We also recorded strong agreement on special assistance for individuals with FASD within the mainstream schools, so that they can benefit from inclusive education. Authors have advocated for specialised FASD classrooms and inclusive classrooms with FASD support [10].

Increasing the education and raising awareness around FASD, especially on the dangers of consuming alcohol during pregnancy in educational settings, clinics, communities and public places should also form a part of an FASD prevention policy. The need for education and awareness programmes is accentuated as research supports the efficacy of awareness intervention, particularly in areas where they are low [26]. These findings echo the urgent call for awareness in South Africa on the dangers of prenatal alcohol exposure and the overwhelming consequence of FASD on the lives of children, families and communities [3]. Comprehensive awareness and health promotion efforts correspond to the first level of prevention of the four-part model of prevention for FASD [44]. Awareness is also a core part of FASD policy strategies in Australia and Canada [34, 36, 37]. Therefore, awareness should be an integral part of a policy guiding the prevention and management of FASD [23].

The first step toward managing FASD in a proposed policy should include the screening of all children who are known to have been prenatally exposed to alcohol. This targeted screening has also been supported by another study [45]. The respondents, nevertheless, cautioned that it is dangerous to screen and diagnose without adequate care and management services. We also found a high agreement with the idea that an FASD policy should include programmes for screening for alcohol use at clinics and hospitals and proper documentation of the history of alcohol use. Therefore, healthcare providers must be equipped with the necessary skills to have informed discussions on alcohol use with women [44, 46]. Discussing alcohol consumption and documenting its history also forms part of the recommendations of the Canadian framework for action on FASD, and the Australian action plans for FASD [34, 36, 37].

Training professionals (health care, social service, criminal justice and judiciary) regarding the prevention, screening, identification, diagnosis and management of FASD should also be considered in FASD policy. The continuous education and training of the professionals to improve their knowledge regarding FASD have also been advocated by some authors [47–51]. The training of these professionals is important because the prevention and management of FASD require a highly skilled multidisciplinary team [12–15]. Training is also essential as it is difficult to manage FASD because of its negative educational, health and social outcomes [52]. The training of parents and caregivers on the needs and how to care for individual with FASD should also be considered when designing policies to inform FASD programmes and interventions. Therefore, training is important, as the needs of individuals with FASD are enormous and parents/caregivers may not have sufficient knowledge of raising individuals with FASD [53]. Policies for FASD in other parts of the world have also considered the training of professionals in addressing FASD [34, 36, 37].

Our findings indicated the inclusion of support to the parents, caregivers, and individuals with FASD in policy which is relevant. These findings reflect one of the practice points for primary healthcare providers [PHCP], stipulating that “PHCP should be aware of FASD support services in their community and refer families to educational and family supports early” [54]. These findings also correspond to level four of the four-part model for FASD – postpartum support for new mothers and supports for child assessments and development [44]. The need for more support for families raising children with FASD has been indicated in a study [53]. Furthermore, Kapasi [55] found caregivers of individuals with FASD to have challenges, indicating the need for support. Challenges such as extra responsibility, difficulty in keeping a daily routine, feeling stigmatised and isolated, managing a child with antisocial behaviour problems and working with a child with diminished executive functioning [55]. Moreover, policies developed in Australia and Canada also advocated for support of the parents, caregivers, and individuals with FASD and community [34, 36, 37].

### Strengths and limitations

We followed a systematic empirical process to elicit information toward formulating the questionnaire. We developed the questionnaire using the findings of four different studies, with the aim of enhancing its content and quality. This process allowed us to gather credible information to be included in the questionnaire as all the participants had expertise/knowledge regarding FASD.

The questionnaire was also shared with local and international experts on FASD for comments and improvement through a discursive process to ensure the essential parts were covered before it was used for the Delphi approach. The use of the Delphi approach provides the opportunity for participants to provide their opinions without fear of having differing views with others. This approach led to a wide range of opinions, which improved the guideline. The Delphi approach provides a strong basis for the construct validity of the questionnaire with participants being able to validate their initial responses and identify areas of uncertainty. The Delphi approach used in this study also allows controlled feedback, which provided the researchers with an opportunity to organise information, if applicable remove duplicates before exchanging the information with the experts.

The purposive and snowballing sampling method used may not have provided the true representation of all the individuals who are knowledgeable or working in the area of FASD. Therefore, this method could have limited the conclusion drawn from the study. A lack of participation from criminal justice and judicial professionals is also a limitation of this study, as their perspectives have been considered essential in the literature. Additionally, we did not include individuals with FASD and their biological or foster parents and caregivers.

## Conclusion

FASD in South Africa deserves urgent attention, especially from the government in terms of the policy to coordinate relevant prevention and management programmes and interventions. Developing a comprehensive and inter-sectoral policy to guide programmes and interventions for the prevention and management of FASD could be a good starting point. The guideline developed has the potential to assist the policymakers in the development of a holistic, multi-sectoral and comprehensive policy for FASD or could streamline discussions on an FASD policy in other relevant contexts.

## Supplementary information

**Additional file 1. **a. Agreement with the statements on guiding principle and approach. b. Agreement with statements on education-related proposed prevention measures. c. Agreement with statements on health-related proposed prevention measures. d. Agreement with statements regarding community/social-related proposed prevention measures. e. Agreement with statements regarding education-related proposed management measures. f. Agreement with statements regarding health-related proposed management measures. g. Agreement with statements regarding community/social-related proposed management measures.

## Data Availability

More information on data from this study is available by contacting the corresponding author.

## Abbreviation

ARBD: Alcohol-related birth defects
ARND: Alcohol-related neurodevelopmental disorder
FARR: Foundation for alcohol related research
FAS: Fetal alcohol syndrome
FASD: Fetal alcohol spectrum disorder
NPO: Non-profit Organisation
PFAS: Partial fetal alcohol syndrome
PHCP: Primary healthcare providers
SPSS: Statistical Package for Social Science
WHO: World Health Organization
