# Supplementary material for: A guideline for the prevention and management of Fetal Alcohol Spectrum Disorder in South Africa
Source: BMC Health Serv Res. 2019 Nov 6;19:809. doi: 10.1186/s12913-019-4677-x (PMC6836420; doi:10.1186/s12913-019-4677-x)
Supplement: Supplementary file 1 — Additional file 1. a. Agreement with the statements on guiding principle and approach. b. Agreement with statements on education-related proposed prevention measures. c. Agreement with statements on health-related proposed prevention measures. d. Agreement with statements regarding community/social-related proposed prevention measures. e. Agreement with statements regarding education-related proposed management measures. f. Agreement with statements regarding health-related proposed management measures. g. Agreement with statements regarding community/social-related proposed management measures. [file 12913_2019_4677_MOESM1_ESM.docx]

**Additional file 1a: Agreement with the statements on guiding principle and approach**

|  | Statement | R1 (%) * | R2 (%) * |
| --- | --- | --- | --- |
| 1 | Holistic | **98** | **-** |
| 2 | User- and caregiver-focused | **98** | **-** |
| 3 | Inter-departmental | **98** | **-** |
| 4 | Considerate of needs across the lifespan | **100** | **-** |
| 5 | Collaborative | **98** | **-** |
| 6 | Human rights-based | **93** | **-** |
| 7 | Based on a public health framework | **93** | **-** |
| 8 | Culturally diverse and culturally sensitive | **100** | **-** |
| 9 | Evidence-based | **98** | **-** |
| 10 | Woman/family centred | **84** | **-** |
| 11 | Clear about referral pathways | **100** | **-** |
| 12 | Designed to avoid victim blaming | **95** | **-** |
| 13 | Cost-effective | **93** | **-** |
| 14 | Driven by behavioural economics | **79** | **81** |
| 15 | Family-centred | **-** | **95** |
| 16 | Women-centred | **-** | **68** |
| 17 | Government-driven/led | **-** | **68** |
| 18 | Address social determinants of health contributing to FASD | **-** | **95** |
| 19 | Designed to give special consideration to the hot spot (rural area) | **-** | **84** |
| 20 | Incorporate home-based care | **-** | **83** |
| 21 | Considerate of input from individuals with FASD and their families | **-** | **94** |
| 22 | Designed to promote responsible parenting | **-** | **98** |
| 23 | Designed to avoid gender-focused interventions | **-** | **66** |
| 24 | Designed assign responsibilities | **-** | **78** |

R1 – Round 1; R2 – Round 2; *Represents ‘agree and strongly agree’

**Additional file 1b: Agreement with statements on education-related proposed prevention measures**

|  | Statement | R1 (%) * | R2 (%) * |
| --- | --- | --- | --- |
| 1 | Enhance awareness of the dangers of drinking alcoholic beverages during pregnancy in schools including colleges and universities | **100** | **-** |
| 2 | Assist individuals with alcohol-use problems in educational settings to access treatment | **98** | **-** |
| 3 | Address barriers to access treatment for alcohol-related problems in educational settings | **98** | **-** |
| 4 | Address stigma associated with alcohol abuse in educational settings | **93** | **-** |
| 5 | Facilitate the training of teachers re the FASD prevention/awareness programme | **98** | **-** |
| 6 | Facilitate the development and implementation of FASD awareness programmes in schools (including colleges and universities) | **95** | **-** |
| 7 | Facilitate the use of peer education re the FASD awareness programme in schools | **91** | **-** |
| 8 | Promote a healthy lifestyle in schools through sport and other extra-curricular activities | **91** | **-** |
| 9 | Make school events alcohol-free | **81** | **78** |
| 10 | Discourage the establishment of the liquor stores in the proximity of schools | **88** | **-** |
| 11 | Promote education of young individuals about healthy pregnancy in schools | **96** | **-** |
| 12 | Facilitate the teaching of responsible parenthood in educational settings | **-** | **90** |
| 13 | Facilitate the teaching of safe sex practices in educational settings | **-** | **88** |

R1 – Round 1; R2 – Round 2; *Represents ‘agree and strongly agree’

**Additional file 1c: Agreement with statements on health-related proposed prevention measures**

|  | Statement | R1 (%) * | R2 (%) * |
| --- | --- | --- | --- |
| 1 | Facilitate screening for alcohol use in clinics and hospitals | **88** | **-** |
| 2 | Encourage proper documentation of the alcohol history for women, especially pregnant women | **95** | **-** |
| 3 | Facilitate the inclusion of FASD prevention as a part of health promotion activities in clinics and hospitals | **100** | **-** |
| 4 | Facilitate the education of individuals and couples on the dangers of drinking alcoholic beverages during pregnancy in the pre-conception clinic | **100** | **-** |
| 5 | Facilitate the education of individuals and couples on the dangers of drinking alcoholic beverages during pregnancy in the reproductive clinic | **100** | **-** |
| 6 | Encourage the use of visible posters and pamphlets for FASD prevention campaigns in all clinics and hospitals | **95** | **-** |
| 7 | Facilitate the training of healthcare professionals on FASD prevention | **98** | **-** |
| 8 | Facilitate early and appropriate referral to treatment for individuals (including women) with alcohol misuse issues | **100** | **-** |
| 9 | Empower health professionals with the skills to counsel and ask questions about alcohol use in a safe and appropriate way | **100** | **-** |
| 10 | Promote the use of contraceptives to avoid unplanned pregnancy | **95** | **-** |
| 11 | Assist parents of individuals with FASD to avoid having another child with FASD | **-** | **93** |

R1 – Round 1; R2 – Round 2; *Represents ‘agree and strongly agree’

**Additional file 1d: Agreement with statements regarding community/social-related proposed prevention measures**

|  | Statement | R1 (%) * | R2 (%) * |
| --- | --- | --- | --- |
| 1 | Facilitate educating public awareness re the dangers of alcohol abuse | **95** | **-** |
| 2 | Facilitate the education of all people in the community re the dangers of drinking alcohol during pregnancy | **100** | **-** |
| 3 | Facilitate the education of individuals and couples re the dangers of drinking alcohol during pregnancy | **100** | **-** |
| 4 | Encourage the use of community groups for FASD prevention (education and awareness) | **95** | **-** |
| 5 | Facilitate the training of the community health/community-based workers and youth care/social workers re FASD prevention | **100** | **-** |
| 6 | Facilitate early intervention and assistance for individuals with alcohol use problems in the community | **100** | **-** |
| 7 | Facilitate the creation of social programmes such as skills training and empowerment programmes for women in the community | **98** | **-** |
| 8 | Encourage awareness and education re FASD in the workplace, rural and urban areas and farming communities | **100** | **-** |
| 9 | Promote the use of multimedia such as posters, adverts, pamphlets, TV, social media and road shows re FASD awareness in the communities | **100** | **-** |
| 10 | Promote enforcement of liquor laws and regulation of shebeens to control accessibility and availability of alcohol in the community | **95** | **-** |
| 11 | Provide access to treatment for people with alcohol-use problems in the community | **100** | **-** |
| 12 | Provide smooth aftercare and community reintegration for people who have attended alcohol rehab | **95** | **-** |
| 13 | Promote afterschool activities in the community to prevent early exposure of adolescents to alcohol | **100** | **-** |
| 14 | Discourage all advertisements that link alcohol to sport/other popular community events/activities | **81** | **71** |
| 15 | Mandate labels on alcohol containers to contain information re the dangers of drinking alcoholic beverages during pregnancy | **95** | **-** |
| 16 | Mandate that liquor stores have warning signs regarding alcohol and pregnancy | **98** | **-** |
| 17 | Enable the creation of support groups for individuals with alcohol misuse issues in the community | **95** | **-** |
| 18 | Facilitate the training of the community and religious leaders re FASD prevention | **95** | **-** |
| 19 | Promote collaboration and the use of non-profit organisations (NPO) for FASD prevention | **98** | **-** |
| 20 | Utilise community and religious leaders to increase FASD awareness in their communities | **95** | **-** |
| 21 | Promote the expansion and adoption of NPO evidence-based interventions for prevention in the community | **98** | **-** |
| 22 | Assist families to support individuals with alcohol-use problems | **98** | **-** |
| 23 | Facilitate the curbing of opening hours, increasing the price of alcohol and the legal age for drinking alcohol, and facilitate the limiting of liquor licences | **-** | **73** |
| 24 | Promote intervention services for mothers who have a child with FASD in the community | **-** | **100** |

R1 – Round 1; R2 – Round 2; *Represents ‘agree and strongly agree’

**Additional file 1e: Agreement with statements regarding education-related proposed management measures**

|  | Statement | R1 (%) * | R2 (%) * |
| --- | --- | --- | --- |
| 1 | Facilitate the development of a curriculum that accommodates individuals with FASD | **93** | **-** |
| 2 | Facilitate the training of teachers re the classroom management for individuals with FASD | **95** | **-** |
| 3 | Promote skilled schools for learners with learning disabilities (including individuals with FASD) who are not benefiting from formal education | **98** | **-** |
| 4 | Make provision for special assistance for individuals with FASD within mainstream schools | **100** | **-** |
| 5 | Facilitate the creation of the special schools for learners with a learning disability (including individuals with FASD) who are not benefiting from mainstream schooling | **93** | **-** |
| 6 | Facilitate the education of parents re the needs and management of individuals with FASD | **98** | **-** |

R1 – Round 1; R2 – Round 2; *Represents ‘agree and strongly agree’

**Additional file 1f: Agreement with statements regarding health-related proposed management measures**

|  | Statement | R2 (N) | R2 (%) * |
| --- | --- | --- | --- |
| 1 | Facilitate capacity building re diagnosis among health professionals | **98** | **-** |
| 2 | Facilitate FASD screening for all children who are known to have been prenatally exposed to alcohol | **89** | **-** |
| 3 | Make provision for diagnostic services for individuals | **95** | **-** |
| 4 | Promote diagnosis for school children, adolescents and adults to reduce rates of people who are left undiagnosed or misdiagnosed | **86** | **-** |
| 5 | Promote appropriate referral pathways to services after diagnosis | **98** | **-** |
| 6 | Facilitate the creation of diagnostic centres in clinics, hospitals and communities | **91** | **-** |
| 7 | Facilitate the creation of national surveillance for FASD via reports from health professionals | **88** | **-** |
| 8 | Make provision for integrated and individualised medical services for individuals with FASD | **88** | **-** |
| 9 | Encourage routine consideration of FASD in the diagnosis and management of mental illness and developmental disorders | **93** | **-** |
| 10 | Make provision for integrated medical services for individuals with FASD | **-** | **88** |
| 11 | Make provision for individualised medical services for individuals with FASD | **-** | **81** |

R1 – Round 1; R2 – Round 2; *Represents ‘agree and strongly agree’

**Additional file 1g: Agreement with statements regarding community/social-related proposed management measures**

|  | Statement | R1 (%) * | R2 (%) * |
| --- | --- | --- | --- |
| 1 | Provide skills training and empowerment programmes for those who need it among individuals with FASD | **95** | **-** |
| 2 | Facilitate appropriate employment opportunities for individuals with FASD | **91** | **-** |
| 3 | Facilitate the training of community health workers/community-based workers/ youth care workers/ social workers and professionals within judiciary system re FASD management | **93** | **-** |
| 4 | Facilitate the training of the biological and foster parents/caregivers regarding the management of FASD | **98** | **-** |
| 5 | Promote the empowerment of the parents/caregivers of individuals with FASD in the community | **95** | **-** |
| 6 | Promote the establishment of support systems for biological and foster parents/caregivers and individuals with FASD in the community | **95** | **-** |
| 7 | Promote the referral of parents and individuals with FASD to appropriate services | **98** | **-** |
| 8 | Make provision for effective counselling services for parents and individuals with FASD | **98** | **-** |
| 9 | Encourage family/community support for individuals with FASD | **98** | **-** |
| 10 | Provide support for individuals with FASD in child protection/foster care and criminal justice system | **98** | **-** |
| 11 | Facilitate the creation of structure and supportive environment at home, school and beyond | **93** | **-** |
| 12 | Promote grant/social welfare for individuals with FASD | **-** | **49** |
| 13 | Make provision for adequate information about individuals with for the adoptive parents | **-** | **100** |

R1 – Round 1; R2 – Round 2; *Represents ‘agree and strongly agree’
